# Editorialets

**Published:** 1908-02

**Authors:** 


### Editorialets.

ANOTHER ONE SAYS: "Find enclosed 50 cents, which payes [sic] me up, and discontinue; money is so scarce," etc., etc.

---

ANOTHER ONE SENDS a V (five years in advance); and another, still, $3 in advance; and I have one who is paid up to 1914. They all say, "Long may she wave!" (except the 50-cent fellow). Makes me feel good.

---

ONE VALUED SUBSCRIBER WRITES: "Dollars are mighty scarce in this neck of the woods, but here is one for 'Betty and the baby,' which I send with a kiss (for the baby), and God bless you (and 'Betty') and the faithful 'Red Back.'"

DR. C. W. SKIPPER, of Lovelady, Texas, son and partner of Dr. R. W. Skipper, a graduate of the Medical Department University of Texas, 1907, has accepted a position as Interne in the City Hospital at Shreveport, and has removed to that city. Best wishes attend him!

---

THE *American Journal of Clinical Medicine* for January is a splendid production, both as to extent and variety of contents, and mechanical get up. It does great credit to its founder and editor-proprietor, "Square Deal Abbott," as we shall hereafter call him. Moreover, it is embellished with a large portrait of Dr. Abbott, in colors, and his autograph—"Yours for a Square Deal, W. C. Abbott." I have framed it and hung it over my desk, where it will serve as an inspiration to renewed energy, whenever I experience, as I sometimes do, that tired feeling and sigh, "What's the use?"

